# TACC2 (transforming acidic coiled‐coil protein 2) in breast carcinoma as a potent prognostic predictor associated with cell proliferation

**DOI:** 10.1002/cam4.736

**Published:** 2016-06-22

**Authors:** Yoshiaki Onodera, Kiyoshi Takagi, Yasuhiro Miki, Ken‐ichi Takayama, Yukiko Shibahara, Mika Watanabe, Takanori Ishida, Satoshi Inoue, Hironobu Sasano, Takashi Suzuki

**Affiliations:** ^1^ Department of Anatomic PathologyTohoku University Graduate school of MedicineSendaiJapan; ^2^ Pathology and HistotechnologyTohoku University Graduate school of MedicineSendaiJapan; ^3^ Department of Anti‐Aging MedicineGraduate School of MedicineThe University of TokyoTokyoJapan; ^4^ Department of Geriatric MedicineGraduate School of MedicineThe University of TokyoTokyoJapan; ^5^ Department of PathologyTohoku University HospitalSendaiJapan; ^6^ Surgical OncologyTohoku University Graduate School of MedicineSendaiJapan; ^7^ Division of Gene Regulation and Signal TransductionResearch Center for Genomic MedicineSaitama Medical UniversityHidaka, SaitamaJapan; ^8^ Functional BiogerontologyTokyo Metropolitan Institute of GerontologyTokyoJapan

**Keywords:** Breast cancer, prognostic markers, proliferation

## Abstract

Transforming acidic coiled‐coil protein 2 (TACC2) belongs to TACC family proteins and involved in a variety of cellular processes through interactions with some molecules involved in centrosomes/microtubules dynamics. Mounting evidence suggests that TACCs is implicated in the progression of some human malignancies, but significance of TACC2 protein in breast carcinoma is still unknown. Therefore, in this study, we examined the clinical significance of TACC2 in breast carcinoma and biological functions by immunohistochemistry and in vitro experiments. Immunohistochemistry for TACC2 was performed in 154 cases of invasive ductal carcinoma. MCF‐7 and MDA‐MB‐453 breast carcinoma cell lines were transfected with small interfering RNA (siRNA) for TACC2, and subsequently, cell proliferation, 5‐Bromo‐2′‐deoxyuridine (BrdU), and invasion assays were performed. TACC2 immunoreactivity was detected in 78 out of 154 (51%) breast carcinoma tissues, and it was significantly associated with Ki‐67 LI. The immunohistochemical TACC2 status was significantly associated with increased incidence of recurrence and breast cancer‐specific death of the patients, and multivariate analyses demonstrated TACC2 status as an independent prognostic factor for both disease‐free and breast cancer‐specific survival. Subsequent *in vitro* experiments showed that TACC2 significantly increased the proliferation activity of MCF‐7 and MDA‐MB‐453. These results suggest that TACC2 plays an important role in the cell proliferation of breast carcinoma and therefore immunohistochemical TACC2 status is a candidate of worse prognostic factor in breast cancer cases.

## Introduction

It is well‐known that breast cancer is one of the most common malignancies in women worldwide. Invasive breast cancer is regarded as a disease that metastasizes in an early phase 1, and clinical outcome of the cases is markedly influenced by proliferative activity of the carcinoma cells 2. Indeed, a multitude of prognostic factors identified in cases with breast cancer have been demonstrated to be directly or indirectly correlated with carcinoma cells’ proliferation. Adjuvant therapy such as endocrine therapy (tamoxifen, aromatase inhibitor, fulvestrant *etc*.) and chemotherapy is frequently used after the surgical treatment, however, it is true that a part of these carcinomas obtain clinical resistance and recur despite the therapy 3, 4. Therefore, it is very valuable to evaluate clinical and biological markers in the breast cancer cases to predict their recurrence after surgery and indications of additional therapies appropriately.

Transforming acidic coiled‐coil proteins (TACCs) are known to be the centrosomes/microtubules interaction‐associated proteins containing a highly conserved C‐terminal coiled‐coil “TACC domain” in themselves. TACC family contains TACC1‐3 in mammals, and these participated in many cellular processes through the interactions with some molecules involved in centrosome–microtubules dynamics 5. Mounting evidence suggests that TACCs participated in the progression of some carcinomas. For instance, TACC1 was an estrogen‐regulated gene 6 and its amplification was correlated with decrease in survival duration or distant recurrence in breast cancer 7. While, TACC3 was suggested a potential glioblastoma multiforme oncogene 8 and an association with cervical tumorigenesis 9. On the other hand, TACC2 has been demonstrated to promote androgen‐mediated growth in the prostate cancer 10, but previous evidences of TACC2 in breast cancer were inconsistent and its significance remains largely unclear. TACC2 was mentioned as a putative breast tumor suppressor 11, whereas Cheng et al. (2010) reported that high expression of TACC2 mRNA in tumor tissue was associated with shorter disease‐free survival of breast carcinoma patients by real‐time PCR analysis 12. It may be partly due to that significance of TACC2 protein has not tested in the breast carcinoma tissues, to the best of our knowledge. Therefore, in our study, we examined TACC2 in breast carcinoma by immunohistochemistry and in vitro experiments to investigate its clinical significance and biological functions. Since TACC2 consists of two isoforms (i.e., major 4.2 kb transcript and minor 9.7 kb transcript) and the major isoform is predominantly expressed in the breast 13, we examined the major isoform of TACC2 in the present in vitro studies.

## Materials and Methods

### Patients and tissues

One hundred and fifty‐four specimens of invasive ductal carcinoma (IDC), not other specified were obtained from Japanese female cases who underwent surgical treatment from 1995 to 1999 (*n* = 56) and 2007 to 2008 (*n* = 98) in the Tohoku University Hospital (Sendai, Japan) (range of age; 27–87). Review of the charts revealed that 120 cases received adjuvant endocrine therapy, and tamoxifen and aromatase inhibitor were mainly used in the former and later periods, respectively. In addition, 88 patients received chemotherapy before (*n* = 17) and/or after the surgery. The clinical outcome of the cases was evaluated by disease‐free and breast cancer‐specific survival in this study. Disease‐free survival was defined as the duration from surgery to the time of the first locoregional recurrence or first distant metastasis within the follow‐up time after surgery, and breast cancer‐specific survival was defined as the duration from surgery to death from the breast cancer. The mean follow‐up duration was 77 months (range; 3–175 months) in this study.

Research protocols for this study were approved by the Ethics Committee at Tohoku University School of Medicine.

### Immunohistochemistry

Rabbit polyclonal antibody for TACC2 (GTX110516) and mouse monoclonal antibodies for Ki‐67 (MIB1) and androgen receptor (AR; AR441) were purchased from Gene Tex (Hsinchu, Taiwan ROC), DAKO (Carpinteria, CA) and DAKO, respectively. A Histofine Kit (Nichirei Biosciences, Tokyo, Japan), which employs the method of streptavidin‐biotin amplification was used in this study. The visualization of antigen–antibody complex was possible with 3,3′‐diaminobenzidine (DAB) solution and after that counterstained with hematoxylin. As a positive control for TACC2 immunostaining, human prostate carcinoma tissue was used 10. Immunohistochemistry for estrogen receptor (ER) (CONFIRM anti‐ER (SP1)) and progesterone receptor (PR) (CONFIRM anti‐PR (1E2); Roche Diagnostics, Tokyo, Japan) was performed with Ventana Benchmark XT (Roche Diagnostics), and that for HER2 was performed by HercepTest (DAKO). Ki‐67 (MIB1) was purchased from DAKO, and a Histofine Kit (Nichirei Bioscience, Tokyo, Japan) was used for the immunohistochemistry.

### Scoring of immunoreactivity

TACC2 immunoreactivity was detected in the cytoplasm, and cases that had more than 10% of positive carcinoma cells were considered “positive” in this study 10. Immunoreactivity for ER, PR, AR, and Ki‐67 was detected in the nuclei and was evaluated in not less than 1000 carcinoma cells for each case. Subsequently, the percentage of immunoreactivity (labeling index (LI)) was determined, and cases with an ER LI of more than 1% were considered “ER‐positive” according to a previous report 14. HER2 status was evaluated according to the grading system proposed in the HercepTest (DAKO), and strongly circumscribed membrane immunoreactivity of HER2 present in more than 10% carcinoma cells (score 3+) was considered “HER2‐positive”. Also, *HER2* gene amplification was investigated by fluorescence in situ hybridization (FISH) in intermediate scoring (score 2+) cases, and the score 2+ cases that were positive were considered “positive” for HER2 status.

### Cell lines

MCF‐7 was supplied by the Cell Resource Center for Biomedical Research, Tohoku University (Sendai, Japan) and MDA‐MB‐453 was purchased from the American Type Culture Collection (ATCC) (Manassas, VA), respectively. MCF‐7 and MDA‐MB‐453 were cultured in RPMI 1640 (Sigma‐Aldrich, St. Louis, MO) which contains 10% heat‐inactivated fetal bovine serum (FBS; Sigma‐Aldrich) and 1% penicillin–streptomycin (Invitrogen, Carlsbad, CA), and Leiboviz's L‐15 (Gibco BRL, Palo Alto, CA) which contains 10% heat‐inactivated fetal bovine serum (FBS; Sigma‐Aldrich) and 1% penicillin–streptomycin (Invitrogen), respectively. Both MCF‐7 and MDA‐MB‐453 were retro‐authenticated by ATCC with short tandem repeat (STR) profiling and confirmed to be the original cell line (in 2015). All experiments were carried out by both cell lines at passage below 20.

### Small interfering RNA (siRNA) transfection

Two siRNA oligonucleotides for TACC2 used in this study were Stealth RNAi siRNA Duplex Oligoribonucleotides (Invitrogen). The target sequences of siRNA against TACC2 were TACC2HSS116287 (si1): 5′‐GCUAAAGGUACUUACACCUUGAUA‐3′ (sense) and 5′‐UAUCAAAGGUGUAAGUACCUUUAGC‐3′ (anti‐sense) and TACC2HSS116288 (si2): 5′‐CCCUUCAGAAGCGAUUGAAAUUACA‐3′ (sense) and 5′‐UGUAAUUUCAAUCGCUUCUGAAGGG‐3′ (anti‐sense). Universal RNAi Negative Control Duplexes (NC; Sigma–Aldrich) were also used as the negative control siRNAs. The siRNAs (10  nmol/L) were transfected with the Lipofectamine RNAiMAX Transfection Reagent (Invtrogen) in accordance with the manufacturer's protocol.

### Real‐time PCR

Total RNA was extracted from cultured cells using the TRIzol Reagent (Invitrogen), subsequently complementary DNA (cDNA) was synthesized using an RT kit (Superscript II preamplification system) (Gibco BRL). The LightCycler System (Roche Diagnostics) was used to semi‐quantify the mRNA expression levels by real‐time PCR. The primer sequences used in real‐time PCR were as follows: TACC2 major isoform (NM_206860): forward, 532–549 and reverse, 671–689; and ribosomal protein L13a (RPL13A) (NM_012423): forward, 487–509 and reverse, 588–612. The DNA binding dye SYBR Green I (Roche Diagnostics) was used for the detection of PCR products, and the relative mRNA level in each sample was calculated as the ratio of RPL13A in this study.

### Immunoblotting

The whole‐cell proteins from MCF‐7 and MDA‐MB‐453 cells were extracted using M‐PER mammalian protein extraction reagent (Thermo Fisher Scientific Pierce Biotechnology, Rockford, IL). The lysate proteins (10  *μ*g) were subjected to SDS–PAGE (10% acrylamide gel). Following SDS–PAGE, the proteins were transferred onto Hybond PVDF membranes (GE Healthcare, Buckinghamshire, UK). Primary antibody used was anti‐TACC2 antibody (Gene Tex), ER (6F11, Novocastra, Newcastle upon Tyne, UK) and AR (AR441, DAKO). In addition, anti‐*β*‐actin (AC‐15, Sigma‐Aldrich) antibody was used as an internal control. Antibody‐protein complexes on the blots were detected using ECL‐Plus Western Blotting Detection Reagents (GE Healthcare), and the protein bands were visualized using ChemiDoc^™^ XRS+ system (Bio‐Rad Laboratories, Inc., Hercules, CA).

### Cell proliferation assay

After 24 h, transfection of the MCF‐7 and MDA‐MB‐453 cells with TACC2 siRNA, the medium was changed to phenol red‐free RPMI 1640 medium containing 10% dextran‐coated charcoal–FBS. After 72 h, the transfection status of cell proliferation was measured according to the WST‐8 (2‐(2‐methoxy‐4‐nitrophenyl)‐3‐(4‐nitrophenyl)‐5‐(2,4‐disulfophenyl)‐2H‐tetrazolium, monosodium salt) method using Cell Counting Kit‐8 (Dojindo Molecular Technologies, Kumamoto, Japan).

### BrdU assay

The percentage of cells in the cell cycle was evaluated with the 5‐Bromo‐2′‐deoxyuridine (BrdU) Labeling and Detection Kit II (Roche Diagnostics) in MCF‐7 and MDA‐MB‐453 cell lines. Briefly, the cells were grown on chamber slides at a density of 1 × 10^6^/mL. After 24 h of incubation, they were transfected with siRNA for TACC2 or control siRNA. The cells were incubated with control siRNA or siRNA for TACC2 for 24 h. Cells were then fixed after BrdU incorporation using ethanol fixative at −25°C. After primary and secondary labeling with anti‐BrdU working solution, BrdU detection was performed according to the manufacturer's protocol. The percentage of BrdU‐positive cells was evaluated in no less than 1000 cells three times 15.

### Wound‐healing assay

The migration property of MCF‐7 and MDA‐MB‐453 cells transfected with siRNA for TACC2 was evaluated by wound scratch healing assay. Briefly, the confluent cell layer was scratched using a sterile plastic P‐200 pipette tip after transfection and incubated for 48  h. The migration area was evaluated using the MultiGauge v3.1 Software (Fuji Photo Film, Tokyo, Japan), and the relative migration area was then calculated as the ratio of that in the control cells transfected with negative control (NC) in this study 16.

### Statistical analysis

In the immunohistochemistry, Student's *t*‐test or a cross‐table using the chi‐squared test were used to evaluate TACC2 status and clinicopathological factors. Disease‐free and breast cancer‐specific survival curves were generated by the Kaplan–Meier method. Then, statistical significance was calculated using the log‐rank test. Univariate and multivariate analyses were evaluated by a proportional hazard model (Cox). *P *<* *0.05 and 0.05 ≤ *P *<* *0.10 were considered significant and borderline‐significant, respectively, in this study 17. In the in vitro experiments, the statistical analyses were performed using one‐way ANOVA and Fisher's protected least significant difference. The statistical analyses were performed using the StatView 5.0J software (SAS Institute, Cary, NC).

## Results

### Immunolocalization of TACC2 in human breast carcinoma

**Figure 1:**
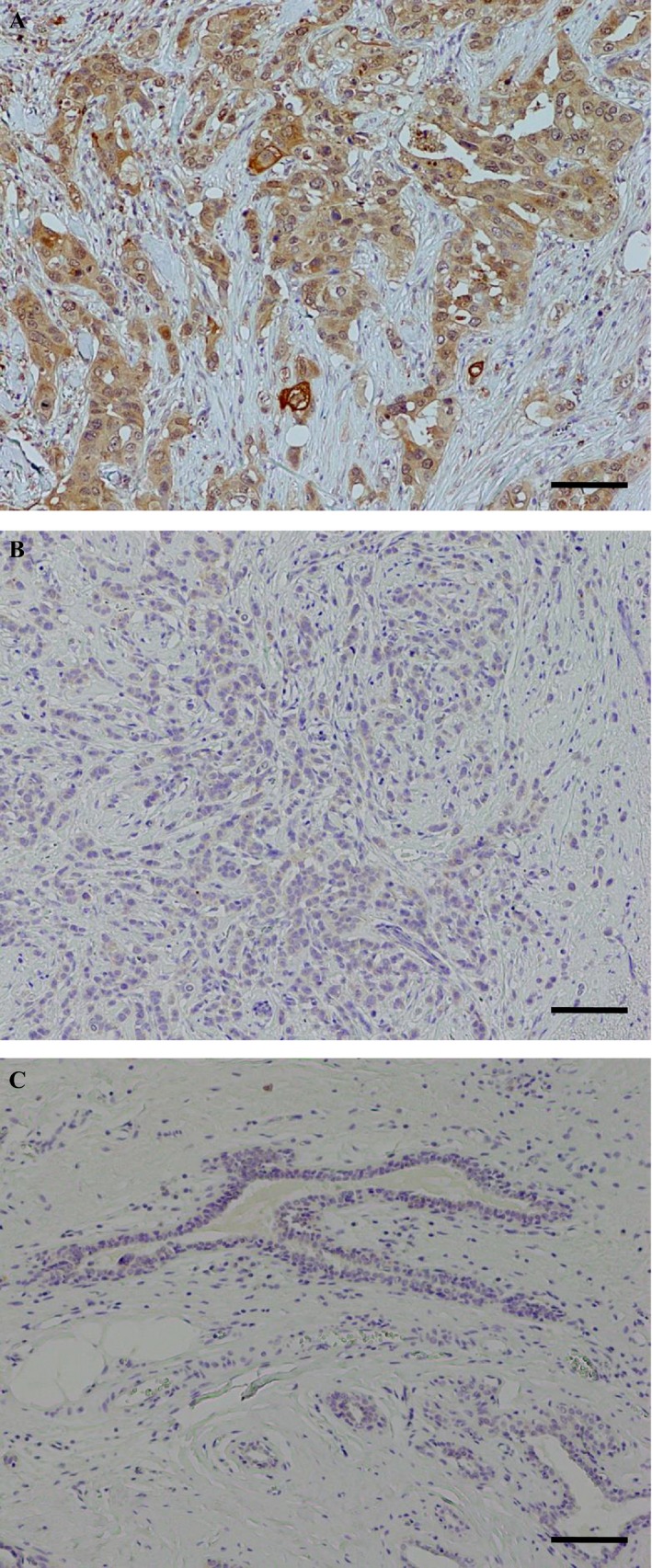
Immunolocalization of TACC2 in human breast carcinoma. (A) TACC2 was immunolocalized in the cytoplasm of carcinoma cells. (B) TACC2‐negative breast carcinoma case. (C) TACC2 immunoreactivity was negative in the morphologically normal mammary gland or stroma. Bar = 100 *μ*m, respectively.

TACC2 immunoreactivity was detected in the cytoplasm of breast carcinoma cells (Fig. 1A, B), while it was negative in the morphologically normal glands or stroma (Fig. 1C). Associations between TACC2 immunoreactivity and various clinicopathological parameters in the breast carcinoma cases were summarized in Table 1. The number of TACC2‐positive cases was 78 out of 154 (51%) in this study. Immunohistochemical TACC2 status was significantly associated with Ki‐67 LI (*P *=* *0.047), but no significant association was detected between TACC2 status and other clinicopathological parameters examined, such as patients’ age, menopausal status, stage, status of neoadjuvant chemotherapy, pathological tumor factor (pT), lymph node metastasis, histological grade, ER status, PR status, AR status, and HER2 status.

**Table 1:** Association between TACC2 immunohistochemical status and clinicopathological parameters in 154 breast carcinoma cases

	TACC2 status		
	+(*n* = 78)	−(*n* = 76)	*P*‐value
Age^a^ (years)	56.1 ± 1.5	57.4 ± 1.3	0.53
Menopausal status			
Premenopausal	30	25	
Postmenopausal	48	51	0.47
Stage			
I	36	37	
II	29	27	
III	13	12	0.95
NAC			
Received	11	6	
Not received	67	69	0.23
Pathological T factor (pT)			
pT1	46	46	
pT2‐4	32	40	0.84
Lymph node metastasis			
Positive	32	28	
Negative	46	48	0.59
Histological grade			
1 (well)	25	24	
2 (moderate)	31	36	
3 (poor)	22	16	0.52
Estrogen receptor (ER) status			
Positive	63	62	
Negative	15	14	0.90
PR status			
Positive	54	55	
Negative	24	21	0.67
AR LI^a^ (%)	29.7 ± 3.0	32.8 ± 3.0	0.46
HER2 status			
Positive	13	10	
Negative	65	66	0.54
Ki‐67 LI^a^ (%)	17.5 ± 1.6	13.4 ± 1.3	**0.047**

a Data are presented as mean ±SEM.

All other values represent the number of cases. Statistical analysis was evaluated by the Student's *t*‐test or a cross‐table using the chi‐squared test. *P‐*values less than 0.05 were considered significant, and described as boldface.

### Association between TACC2 status and clinical outcome of the patients

**Figure 2:**
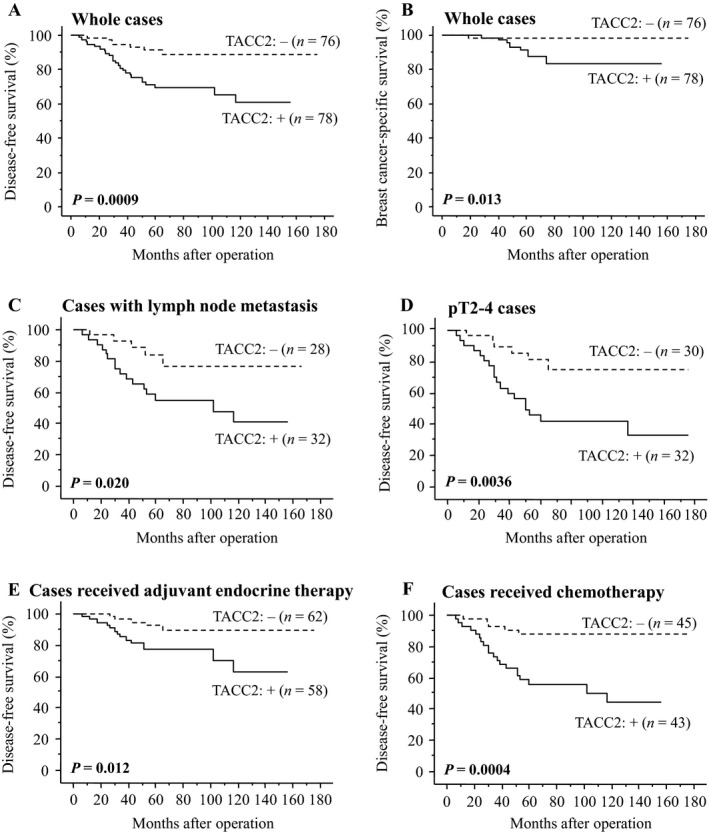
Disease‐free (A, C–F) and breast cancer‐specific survival (B) of breast cancer patients according to TACC2 status by the Kaplan–Meier method. (A, B) whole cases (*n* = 154), (C) cases positive for lymph node metastasis (*n* = 60), (D) pT2‐4 cases (*n* = 62), (E) cases receiving adjuvant endocrine therapy following surgery (*n* = 120), and (F) cases receiving chemotherapy before and/or after the surgery (*n* = 88). The solid line shows TACC2‐positive cases and the dashed line shows TACC2‐negative cases. Statistical analysis was performed using the log‐rank test. *P*‐values <0.05 were considered significant and shown in bold.

As shown in Figure 2A, TACC2 status was significantly associated with an increased incidence of recurrence in breast cancer patients (*P *=* *0.0009 by log‐rank test). Similar trend was shown regardless of the sample collection period (1995–1999 [*n* = 56]: *P *=* *0.039, and 2007–2008 [*n* = 98]: *P *=* *0.011) (data not shown). A significant correlation was also shown between TACC2 status and adverse clinical outcome of these cases (*P *=* *0.013). Significant correlations between TACC2 status and increased risk of recurrence were detected regardless of the lymph node metastasis (cases with lymph node metastasis (*n* = 60): *P *=* *0.020 (Fig. 2C), and cases without lymph node metastasis (*n* = 94): *P *=* *0.035) and pT (pT2‐4 cases (*n* = 62): *P *=* *0.0036 (Fig. 2D), and pT1 cases (*n* = 92): *P *=* *0.047). Moreover, TACC2 status was significantly correlated with recurrence also in the cases who received adjuvant endocrine therapy (*n* = 120; *P *=* *0.012 (Fig. 2E) and the cases who received chemotherapy (*n* = 88; *P *=* *0.0004 (Fig. 2F).

Results of univariate analysis of disease‐free survival using Cox (Table 2), Ki‐67 LI, pT, PR status, lymph node metastasis, TACC2 status, adjuvant endocrine therapy, and chemotherapy were demonstrated to be significant prognostic factors for disease‐free survival and histological grade was detected as the borderline significance. Following multivariate analysis revealed that pT (*P *=* *0.0018), TACC2 status (*P *=* *0.0027), and PR status (*P *=* *0.036) were independent prognostic factors.

**Table 2:** Univariate and multivariate analyses of disease‐free survival in 154 breast cancer patients examined

	Univariate	Multivariate	
Variable	*P‐*value	*P‐*value	Relative risk (95% CI)
Ki‐67 LI			
(0–60)	<**0.0001**	0.12	
pT			
(pT1/pT2‐4)	<**0.0001**	**0.0018**	0.2 (0.1–0.5)
PR status			
(positive/negative)	**0.0011**	**0.036**	0.4 (0.2–0.9)
Lymph node metastasis			
(positive/negative)	**0.0011**	0.36	
TACC2 status			
(+/−)	**0.0021**	**0.0027**	3.8 (1.6–9.2)
Adjuvant endocrine therapy			
(+/−)	**0.0028**		0.63
Chemotherapy (+NAC)			
(+/−)	**0.012**		0.27
Histological grade			
(1,2/3)	*0.056*		
Estrogen receptor (ER) status			
(positive/negative)	0.11		
HER2 status			
(positive/negative)	0.61		

Statistical analysis was evaluated by a proportional hazard model (Cox). Data considered significant (*P *<* *0.05) in the univariate analyses were described as boldface, and these were examined in the multivariate analyses.

As shown in Table 3, univariate analyses for breast cancer‐specific survival revealed Ki‐67, chemotherapy, histological grade, PR status, pT, adjuvant endocrine therapy, ER status, lymph node metastasis, and TACC2 status as significant prognostic variables in these cases, and subsequent multivariate analysis turned out that only PR status (*P *=* *0.016) and TACC2 status (*P *=* *0.040) were independent parameters of these cases in this study.

**Table 3:** Univariate and multivariate analyses of breast cancer‐specific survival in 154 breast cancer patients examined

Variable	Univariate	Multivariate	
	*P‐*value	*P‐*value	Relative risk (95% CI)
Ki‐67 LI			
(0–60)	**<0.0001**	0.27	
Chemotherapy(+NAC)			
(+/−)	**0.0017**	0.46	
Histological grade			
(1,2/3)	**0.0021**		0.41
PR status			
(positive/negative)	**0.0037**	**0.016**	0.1 (0.002–0.6)
pT			
(pT1/pT2‐4)	**0.011**	0.34	
Adjuvant endocrine therapy			
(+/−)	**0.012**	0.47	
Estrogen receptor (ER) status			
(positive/negative)	**0.020**	0.65	
Lymph node metastasis			
(positive/negative)	**0.023**	0.36	
TACC2 status			
(+/−)	0.039	**0.040**	7.1 (1.1–146.6)
HER2 status			
(positive/negative)	0.75		

Statistical analysis was evaluated by a proportional hazard model (Cox). Data considered significant (*P *<* *0.05) in the univariate analyses were described as boldface, and these were examined in the multivariate analyses.

### Effects of TACC2 on cell proliferation and migration in breast carcinoma cells

**Figure 3:**
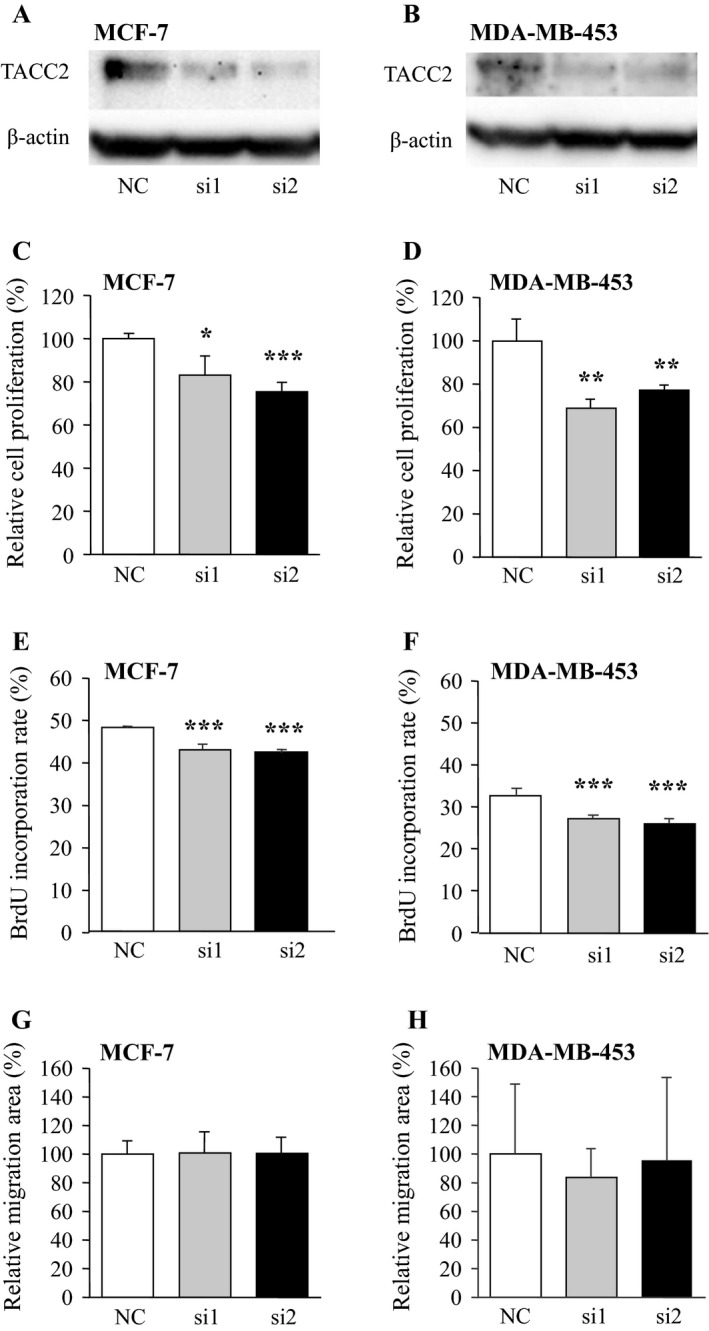
Effects of TACC2 on cell proliferation and invasion in breast carcinoma cells. (A, B) Protein expression of TACC2 major isoform in MCF‐7 (A) and MDA‐MB‐453 (B) cells transfected with TACC2‐specific siRNA (si1 and si2) or negative control siRNA (NC). In the immunoblotting, 10 *μ*g of protein was loaded in each lane, and *β*‐actin immunoreactivity was shown as the internal control. (C, D) Proliferation activity of MCF‐7 (C) and MDA‐MB‐453 (D) cells transfected with siRNA summarized as a ratio (%) compared to that at 0 day after treatment. (E, F)Percentage of BrdU incorporated cells in the MCF‐7 (E) and MDA‐MB‐453 (F). (G, H) Wound‐healing assays in MCF‐7 and MDA‐MB‐453 cells. The relative migration area was evaluated as a ratio (%) compared to the control cells (left bar). Open bar: NC, gray bar: si1; and closed bar: si2. Data are presented as the mean ±S.D. (*n* = 3), respectively. *P <* *0.05, **P <* *0.01, and ***P <* *0.001 compared with negative control group (left bar). The statistical analyses were performed using one‐way ANOVA and Fisher's protected least significant difference.

In order to further evaluate biological functions of TACC2 in human breast carcinoma cells, we transfected specific siRNA for TACC2 in MCF‐7 (ER+ and AR+) and MDA‐MB‐453 (ER‐ and AR+) breast carcinoma cells (Fig. S1). The major isoform of TACC2 protein was detected as a specific band, and it was markedly decreased both in MCF‐7 (Fig. 3A) and MDA‐MB‐453 (Fig. 3B) cells transfected with specific TACC2 siRNA (i.e., si1 and si2) at 24 hours after transfection compared to cells transfected with negative control siRNA (NC).

Effects of TACC2 expression on cell proliferation in breast carcinoma cell lines were summarized in Figures 3C and D. The number of cells was significantly lower in MCF‐7 cells transfected with TACC2 siRNA (*P *<* *0.05 and 0.84‐fold in si1, and *P *<* *0.001 and 0.75‐fold in si2) than the control cells transfected with NC at 72 h after the transfection. Similar tendency was also detected in MDA‐MB‐231 cells under the same condition (*P *<* *0.01 and 0.69‐fold in si1, and *P *<* *0.01 and 0.77‐fold in si2).

When we performed BrdU assay under the same condition, % BrdU incorporation was also significantly lower in MCF‐7 and MDA‐MB‐453 cell lines transfected with TACC2 siRNA (MCF‐7; *P *<* *0.001 and 0.95‐fold in si1, and *P *<* *0.001 and 0.94‐fold in si2 (Fig. 3E), and MDA‐MB‐453; *P *<* *0.001 and 0.94‐fold in si1, and *P *<* *0.001 and 0.93‐fold in si2 (Fig. 3F)) than the control cells transfected with NC.

On the other hand, relative migration areas in MCF‐7 and MDA‐MB‐453 cells transfected with siRNA against TACC2 were not significantly changed compared to their controls in this study (MCF‐7; *P *=* *0.46 in si1 and *P *=* *0.45 in si2 (Fig. 3G), and MDA‐MB‐453; *P *=* *0.19 in si1 and *P *=* *0.45 in si2 (Fig. 3H)).

### Effects of sex steroids on TACC2 expression in breast carcinoma cells

**Figure 4:**
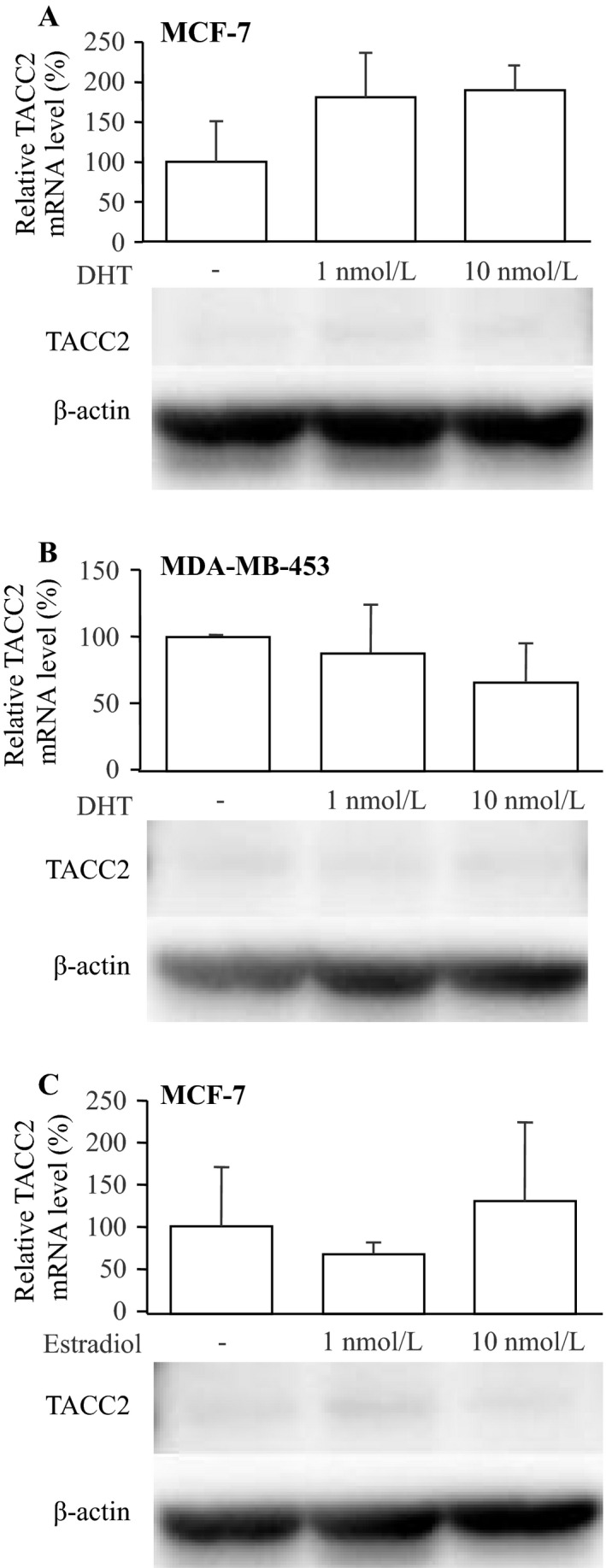
Effects of sex steroids on major TACC2 isoform expression in breast carcinoma cells. (A, B) Effects of DHT on the TACC2 expression in MCF‐7 (A) and MDA‐MB‐453 (B) cells, and (C) Effects of estradiol on the TACC2 expression in MCF‐7 cells. These cells were treated with indicated concentration of dihydrotestosterone (DHT) or estradiol for 24 h. The mRNA level of TACC2 major isoform was examined by real‐time PCR analysis (upper panels). TACC2 mRNA level was evaluated as a ratio of RPL13A mRNA level, and subsequently relative TACC2 mRNA level was summarized as a ratio (%) compared with the basal level (nontreatment). Data are presented as the mean ±S.D. (*n* = 3). The statistical analyses were performed using one‐way ANOVA and Fisher's protected least significant difference. Lower panels summarized the TACC2 protein level by immunoblotting. 10 *μ*g of protein was loaded in each lane, and *β*‐actin immunoreactivity was shown as the internal control.

Bioactive androgen dihydrotestosterone (DHT) did not significantly change the mRNA level of TACC2 major isoform in AR‐positive MCF‐7 (1 and 10 nmol/L DHT; *P *=* *0.14 and 0.19, respectively) (Fig. 4A) and MDA‐MB‐453 (1 and 10 nmol/L DHT; *P *=* *0.34 and *P *=* *0.11, respectively) (Fig. 4B). Also, the TACC2 mRNA was similar level in ER‐positive MCF‐7 cells regardless of the treatment of bioactive estrogen estradiol in this study (1 and 10 nmol/L estradiol; *P *=* *0.14 and 0.19, respectively) (Fig. 4C).

## Discussion

This is a first study which demonstrates clinical significance of TACC2 immunoreactivity in breast carcinoma. In this study, immunoreactivity of TACC2 was detected in 51% of breast carcinoma, while it was negligible in morphologically normal mammary glands. Previously, Cheng et al. showed that TACC2 mRNA level was approximately four times higher in breast carcinoma tissues than the background tissues from the breast cancer patients 12, which is in good agreement with our present results. On the other hand, Takayama et al. reported that TACC2 was overexpressed and its immunoreactivity was positive in 23% of the prostatic carcinoma 10. Our present results suggest that immunoreactivity of TACC2 is increased in a subset of breast carcinoma compared to normal breast tissues, and the relatively wide distribution of immunoreactivity of TACC2 may also indicate biological importance of TACC2 in human breast carcinomas.

In this study, immunoreactivity of TACC2 was positively associated with Ki‐67 LI in breast carcinomas. Ki‐67 antibody recognizes cells in all phases of the cell cycle except G0 (resting) phase, and Ki‐67 LI is well correlated with proliferation activity of breast cancer 2. In addition, results of in vitro studies demonstrated that both MCF‐7 and MDA‐MB‐453 cells transfected with siRNA against TACC2 significantly decreased the cell proliferation and BrdU incorporation rate. TACC2 is strongly concentrated at centrosomes throughout the entire cell cycle 18. Previously, Takayama et al. showed that TACC2 knockdown lead to G2/M accumulation and overexpression of TACC2 increased S‐phase entry after release from G2/M synchronization in prostate carcinoma cells 10. Our present results demonstrated these findings, and it is suggested that TACC2 plays an important role in the cell proliferation of breast carcinoma.

Functional AR‐binding sites were existed in the vicinity of *TACC2* gene, and TACC2 expression was markedly induced by androgen in the prostate carcinoma cells 10. However, in this study, we could not find significant association between TACC2 status and AR LI in breast carcinoma cases (Table 1) or induction of TACC2 mRNA expression by DHT treatment in breast carcinoma cells (Fig. 4A, B). We previously identified 610 DHT‐induced genes in T‐47D breast carcinoma cells by microarray analysis 19, but TACC2 was not included in this list (data not shown). Androgens immensely contribute to growth of prostate cancer through binding with AR, but significance of androgens actions remains largely unclear 20. Previous studies using in vitro techniques demonstrated that DHT predominantly exerted anti‐proliferative effects on mitogenic effects of estrogens in breast carcinoma cells 21, although divergent findings have been reported 22. Moreover, androgen responsive element (ARE)‐dependent transactivation by DHT was markedly suppressed by estrogen in T‐47D breast carcinoma cells, suggesting that androgen actions are, in general, suppressed in breast carcinoma by predominant estrogen actions, even if the carcinoma cells expressed AR and intratumoral DHT level induced significantly 23. Therefore, TACC2 does not necessarily involve in AR signaling and other factors can be important in the regulation of TACC2 expression in the breast carcinoma, different from the prostate carcinoma. For instance, it has been reported that TACC2 was up‐regulated by SMYD2 (SET‐ and MYND‐containing protein 2) gain of function, and it occurred as a result of SMYD2 binding to the TACC2 promoter where it methylated histone H3 at lysine 4 (H3K4) 24. Further examinations are needed to clarify molecular regulatory mechanisms of TACC2 expression in breast carcinoma.

In this study, TACC2 status was significantly associated with recurrence and worse prognosis of female breast cancer cases, and a similar tendency was also shown in the cases which received adjuvant endocrine therapy and/or chemotherapy. Moreover, using multivariate analyses, we demonstrated that TACC2 status turned out an independent worse prognostic factor for both disease‐free and breast cancer‐specific survival. Significance of TACC2 in breast cancer is controversial. TACC2 was identified as a putative breast tumor suppressor 11 and overexpression of TACC2 reverted a malignant phenotype to a benign phenotype 13. On the other hand, single‐nucleotide polymorphisms (SNPs) in TACC2 were significantly associated with a risk of low‐grade breast cancer 25, and TACC2 mRNA expression was significantly correlated with shorter disease‐free survival of breast carcinomas patients 12. An association between TACC2 expression and worse prognosis was also shown in the prostate cancer 10 and infant acute lymphoblastic leukemia 26. Little information is available about association between TACC2 and resistance to adjuvant therapy, however, Takayama et al. showed that TACC2 contributed to hormone‐refractory proliferation in prostate carcinoma 10. Previous studies have also demonstrated that TACC1 was involved in endocrine therapy (tamoxifen and fulvestrant) resistance in breast cancer 27 and TACC3 was associated with chemoresistance (paclitaxel) in uterine cervical cancer 9. Therefore, it is suggested that TACC2 plays an oncogenic role in breast carcinoma by promotion of the cell proliferative activity, and residual carcinoma cells following surgical treatment in TACC2‐positive breast carcinomas may still have the high potential to rapidly grow and/or metastasize, despite the adjuvant therapy.

In summary, immunoreactivity of TACC2 was detected in 51% of breast carcinoma cells, and the TACC2 status was significantly correlated with Ki‐67 LI in these cases. Moreover, TACC2 status was significantly associated with worse prognosis of the cases and it turned out an independent prognostic factor for both the disease‐free and breast cancer‐specific survival. Following in vitro study demonstrated that RNA interference of *TACC2* significantly decreased the proliferation activity of both MCF‐7 and MDA‐MB‐453 cell lines. These results suggest that TACC2 plays an important role in the cell proliferation of breast carcinoma and immunohistochemical TACC2 status may become a potent prognostic factor in these patients.

## Conflict of Interest

We declare that there is no conflict of interest that could be perceived as prejudicing the impartiality of the research reported.

## Supporting information


**Figure S1.** Expression of ER, AR, and TACC2 proteins in MCF‐7 and MDA‐AM‐453 breast carcinoma cells used in this study. A total quantity of 10 *μ*g of protein was loaded in each lane, and *β*‐actin immunoreactivity was shown as the internal control.
