# Supplementary figures and images for: TACC2 (transforming acidic coiled‐coil protein 2) in breast carcinoma as a potent prognostic predictor associated with cell proliferation
Source: Cancer Med. 2016 Jun 22;5(8):1973–82. doi: 10.1002/cam4.736 (PMC4971925; doi:10.1002/cam4.736)

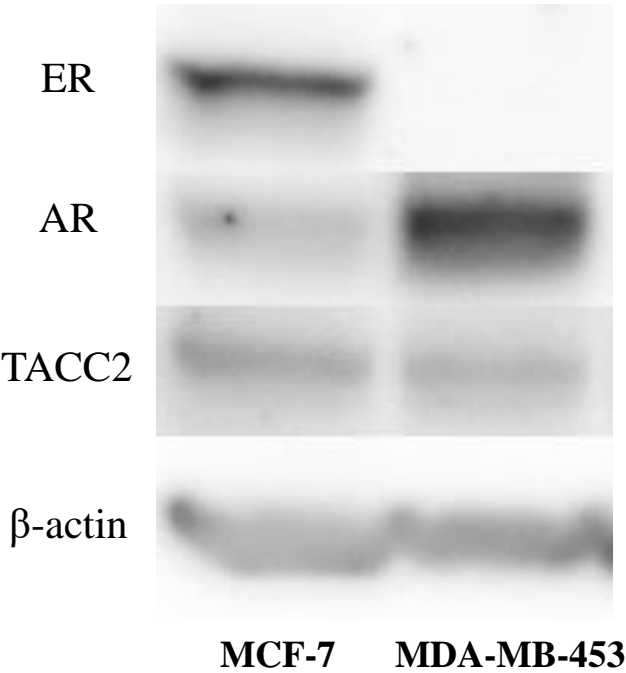

Supplement: Supplementary file 1 — Figure S1. Expression of ER, AR, and TACC2 proteins in MCF‐7 and MDA‐AM‐453 breast carcinoma cells used in this study. A total quantity of 10 μg of protein was loaded in each lane, and β‐actin immunoreactivity was shown as the internal control. [file CAM4-5-1973-s001.pdf]
